# Crystal structure of tetra­kis­[μ-3-carboxy-1-(1,2,4-triazol-4-yl)adamantane-κ^2^ *N* ^1^:*N* ^2^]tetra­fluoridodi-μ_2_-oxido-dioxidodisilver(I)divanadium(V) tetra­hydrate

**DOI:** 10.1107/S2056989019006844

**Published:** 2019-05-17

**Authors:** Ganna A. Senchyk, Andrey B. Lysenko, Eduard B. Rusanov, Kostiantyn V. Domasevitch

**Affiliations:** a Inorganic Chemistry Department, Taras Shevchenko National University of Kyiv, Volodymyrska Street, 64, Kyiv 01033, Ukraine; b Institute of Organic Chemistry, Murmanska Street, 5, Kyiv, 02660, Ukraine

**Keywords:** crystal structure, 1,2,4-triazole, silver(I), vanadium(V) oxofluoride, heterobimetallic complex, hydrogen bonding

## Abstract

The crystal structure of the title mol­ecular complex, [Ag_2_{VO_2_F_2_}_2_(C_13_H_17_N_3_O_2_)_4_]·4H_2_O, supported by the heterofunctional ligand *tr-ad-COOH* [1-(1,2,4-triazol-4-yl)-3-carb­oxy­adamantane] is reported. Four 1,2,4-triazole groups of the ligand link two Ag^I^ atoms, as well as Ag^I^ and V^V^ centres, forming the heterobimetallic coordination cluster {Ag^I^_2_(V^V^O_2_F_2_)_2_(*tr*)_4_}. V^V^ exists as a vanadium oxofluoride anion and possesses a distorted trigonal–bipyramidal coordination environment [VO_2_F_2_N]. A carb­oxy­lic acid functional group of the ligand stays in a neutral form and is involved in hydrogen bonding with solvent water mol­ecules and VO_2_F_2_^−^ ions of adjacent mol­ecules. The extended hydrogen-bonding network is responsible for the crystal packing in the structure.

## Abstract 2

The crystal structure of the title compound is based on the mol­ecular heterobimetallic unit {Ag_2_(VO_2_F_2_)_2_(*tr*)_4_} supported by the 1,2,4-triazole ligand, 1-(1,2,4-triazol-4-yl)-3-carb­oxy­adamantane.

## Chemical context   

Heterometallic hybrids incorporating a metal oxide/oxofluoride matrix are of particular inter­est as they exhibit non-trivial magnetic, luminescent (Cui *et al.*, 2012 ▸), optical and catalytic properties (Dolbecq *et al.*, 2010 ▸). Among the broad range of inorganic anions, vanadium oxofluorides (VOFs) stand out for their large number of types and structural motifs from mono- (Aldous *et al.*, 2007 ▸; Stephens *et al.*, 2005 ▸) to polynuclear (Buchholz *et al.*, 1988 ▸; Ninclaus *et al.*, 1997 ▸) ones in the structure as discrete units or incorporated into coordination frameworks (Welk *et al.*, 2007 ▸; Mahenthirarajah *et al.*, 2008 ▸). The Ag^I^/VOF pair is a non-typical combination for classical coordination chemistry, but materials such as Ag_4_V_2_O_6_F_2_ (Sorensen *et al.*, 2005 ▸; Albrecht *et al.*, 2009 ▸) and Ag_3_VO_2_F_4_ (Chamberlain *et al.*, 2010 ▸) are attractive electrochemically active phases for solid-state batteries.

**Figure 1:**
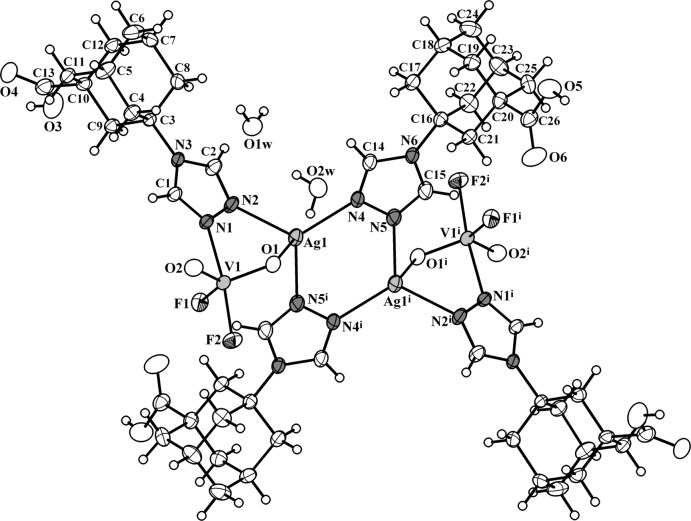
The mol­ecular structure of compound **I**, showing the atomic labelling scheme [symmetry code: (i) –*x*, 1 – *y*, –*z*]. Displacement ellipsoids are drawn at the 30% probability level. The two symmetry-generated water molecules are omitted.

In the present research we introduce a new ligand [*tr-ad-COOH* = 1-(1,2,4-triazol-4-yl)-3-carb­oxy­adamantane], whose 1,2,4-triazole and –COOH donor groups can support the formation of the Ag–V heterometallic coordination cluster. It has recently been shown (Senchyk *et al.*, 2012 ▸) that symmetrical 1,2,4-triazoles can selectively bridge these different metals. Considering a possible step-by-step mechanism, it becomes clear that after the formation of the simplest {Ag_2_(*η* ^2^-*tr*)_2_(*tr*)_2_}^2+^ binuclear fragment, two N atoms remain uncoordinated and have potential for further inter­actions. In aqueous reaction media, vanadium oxofluorides exist in anionic forms with weakly coordinated water mol­ecules that are very labile toward N-donor ligand substitution. Thus, a combination of an Ag^I^–triazole cation and VOF anions lead to the neutral tetra­nuclear {Ag^I^_2_(V^V^O_2_F_2_)_2_(*tr*)_4_} unit, which was found in the structure of the title [Ag_2_(VO_2_F_2_)_2_(*tr-ad-COOH*)_4_]·4H_2_O complex **I** (Fig. 1 ▸).
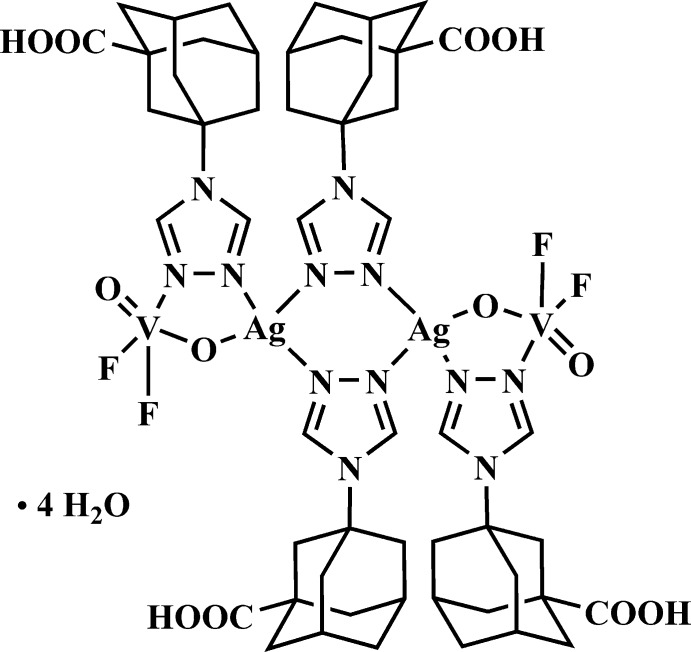


## Structural commentary   

The asymmetric unit of the title compound contains one Ag^I^ cation, one [VO_2_F_2_]^−^ anion, two organic ligands and two solvent water mol­ecules. Two silver ions, two VOF anions and four *tr-ad-COOH* units constitute the mol­ecular tetra­nuclear cluster, which resides across an inversion centre (Fig. 1 ▸). The Ag^I^ atom adopts a distorted tetra­hedral coordination environment [AgN_3_O] with typical Ag—N(triazole) bond lengths [2.230 (3)–2.262 (3) Å; Table 1 ▸] and an elongated Ag—O bond [2.700 (3) Å]. Two 1,2,4-triazole functional groups link two adjacent silver atoms [the Ag⋯Ag^i^ distance is 3.7488 (5) Å; symmetry code: (i) –*x*, −*y* + 1, −*z*], while the other two 1,2,4-triazole groups combine the Ag and V centres [Ag⋯V= 3.5376 (6) Å]. The V^V^ atom possesses a distorted trigonal–bipyramidal coordination environment [VO_2_F_2_N] with short V—O bonds [1.627 (2), 1.628 (2) Å], V—F bonds [1.839 (2), 1.850 (2) Å] and an elongated V—N bond [2.152 (3) Å]. The polyhedra can be more precisely described by the Reedijk’s factor τ (Addison *et al.*, 1984 ▸) of 0.72 (for strict square-pyramidal polyhedra τ = 0 and for trigonal–bipyramidal τ = 1).

As a result, the heterobimetallic unit {Ag^I^_2_(V^V^O_2_F_2_)_2_(*tr*)_4_} is formed. A search in the Cambridge Structural Database (version 5.39, updates of May 2018; Groom *et al.*, 2016 ▸) shows that only three crystal structures containing the Ag^I^/*tr*/V^V^ fragments are known so far (Senchyk *et al.*, 2012 ▸). While considering heterofunctional ligands, {Cu_2_(H*L*)_2_[Mo_4_O_13_]}·2H_2_O (H_2_ *L* = 5-triazole isophthalic acid; Zhu *et al.*, 2012 ▸) is the only known structure where both COO^−^ and triazole groups support the heterometallic Cu⋯Mo connection.

## Supra­molecular features   

**Figure 2:**
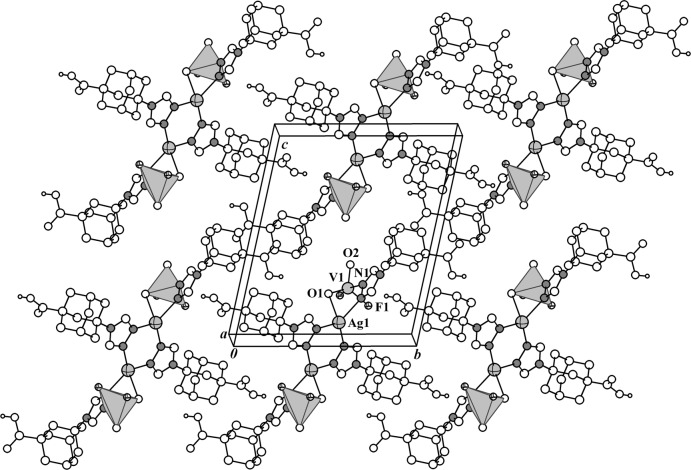
View of the crystal packing of compound **I**. Vanadium oxofluoride anions are shown as polyhedra.

**Figure 4:**
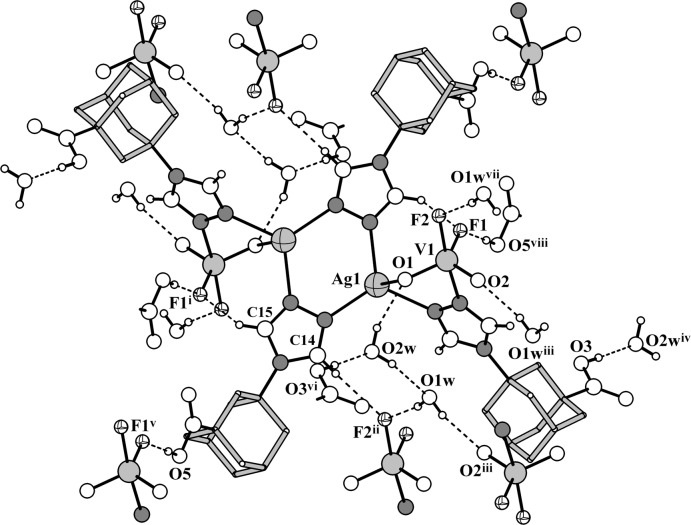
Hydrogen-bonding arrangement in the structure of the title compound. The adamantyl scaffolds are shown in a stick mode omitting the H atoms [symmetry codes: (i) −*x*, 1 − *y*, −*z*; (ii) 1 + *x*, *y*, *z*; (iii) −*x*, 1 − *y*, 1 − *z*; (iv) *x*, 1 + *y*, *z*; (v) 1 + *x*, −1 + *y*, *z*; (vi) *x*, −1 + *y*, *z*; (vii) −1 + *x*, *y*, *z*; (viii) −1 + *x*, 1 + *y*, *z*].

The structure of **I** is characterized by an extended hydrogen-bonding network. The carb­oxy­lic function of the *tr-ad-COOH* ligand remains in a neutral form, being uncoordinated. It is involved in hydrogen bonding that leads to a three-dimensional hydrogen-bonded network (Figs. 2 ▸ and 3 ▸). The nearest environment of the mol­ecular fragment complex involved in hydrogen-bonding inter­actions is shown in Fig. 4 ▸. The corres­ponding geometric parameters are given in Table 2 ▸. One carb­oxy­lic group, as a hydrogen-bond donor, forms a contact with a water mol­ecule O3—H1*O*⋯O2*W* ^iv^ = 2.650 (4) Å [symmetry code: (iv) *x*, 1 + *y*, *z*], while another COOH group, as a hydrogen-bond acceptor, is directed toward the F atom of a {VO_2_F_2_} anion [O5—H2*O*⋯F1^v^ = 2.589 (3) Å; symmetry code: (v) 1 + *x*, −1 + *y*, *z*]. Two water mol­ecules are inter­bonded [O2*W*—H3*W*⋯O1*W* = 2.753 (4) Å] and additionally act as hydrogen-bond donors with O and F atoms from the neighboring {VO_2_F_2_} anions and as hydrogen-bond acceptor (in the case of O2*W* ) with the O3 atom from an adjacent carb­oxy­lic group. Some weak contacts between the triazole C—H groups and F atoms of the VOF anions are also observed.

## Synthesis and crystallization   

1-(1,2,4-Triazol-4-yl)-3-carb­oxy­adamantane (*tr-ad-COOH*) was synthesized in 63% yield by refluxing 3-amino-adamantane-1-carb­oxy­lic acid (Wanka *et al.*, 2007 ▸) (3.00 g, 15.4 mmol) and di­methyl­formamide azine (5.46 g, 38.5 mmol) in the presence of toluene­sulfonic acid monohydrate (0.44 g, 2.3 mmol) as catalyst in DMF (30 ml). Complex **I** was prepared under hydro­thermal conditions as follows. A mixture of AgOAc (16.7 mg, 0.100 mmol), *tr-ad-COOH* (12.4 mg, 0.050 mmol), V_2_O_5_ (9.1 mg, 0.050 mmol) and 5 mL of water with aqueous HF (50%, 150 µL, 4.33 mmol) was added into a Teflon vessel. Then the components were heated at 423 K for 24 h and slowly cooled to room temperature over 50 h, yielding light-yellow prisms of **I** (yield 14.8 mg, 78%).

## Refinement   

Crystal data, data collection and structure refinement details are summarized in Table 3 ▸. The non-H atoms were refined with anisotropic displacement parameters and a soft rigid-bond restraint was applied to C10—C13 in order to improve the refinement stability. C-bound hydrogen atoms were positioned geometrically and refined as riding, with C—H = 0.93 Å (triazole), C—H = 0.97 Å (adamantane CH_2_), C—H = 0.98 Å (adamantane CH) and with *U*_iso_(H) = 1.2*U*_eq_(C). O-bound hydrogen atoms were located in a difference-Fourier map and then refined with O—H = 0.82 Å (carb­oxy­lic) or 0.85 Å (H_2_O) with *U*_iso_(H) = 1.5*U*_eq_(O).

## Supplementary Material

Crystal structure: contains datablock(s) I. DOI: 10.1107/S2056989019006844/zq2246sup1.cif

Structure factors: contains datablock(s) I. DOI: 10.1107/S2056989019006844/zq2246Isup2.hkl

CCDC reference: 1915603

Additional supporting information:  crystallographic information; 3D view; checkCIF report

## Figures and Tables

**Figure 3:**
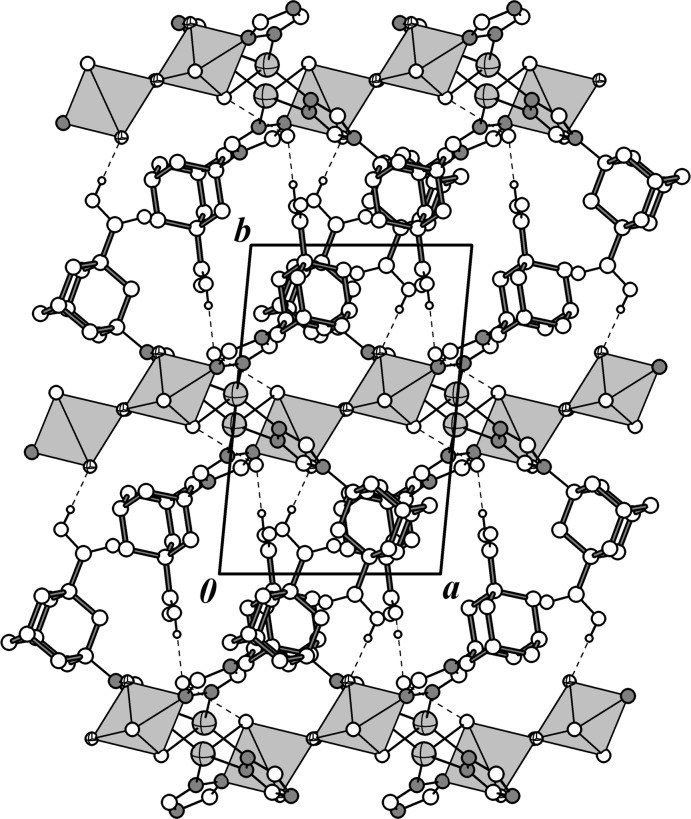
Projection on the *ab* plane showing the crystal packing in the structure. Vanadium oxofluoride anions are shown as polyhedra.

**Table 1:** Selected geometric parameters (Å, °)

Ag1—N4	2.230 (3)	V1—O2	1.628 (2)
Ag1—N2	2.257 (3)	V1—F2	1.839 (2)
Ag1—N5^i^	2.262 (3)	V1—F1	1.850 (2)
Ag1—O1	2.700 (3)	V1—N1	2.152 (3)
V1—O1	1.627 (2)		
			
N4—Ag1—N2	121.35 (11)	O1—V1—F1	122.01 (12)
N4—Ag1—N5^i^	115.91 (10)	O2—V1—F1	122.74 (11)
N2—Ag1—N5^i^	120.21 (11)	F2—V1—F1	86.60 (10)
N4—Ag1—O1	114.33 (9)	O1—V1—N1	87.04 (11)
N2—Ag1—O1	74.30 (9)	O2—V1—N1	87.42 (12)
N5^i^—Ag1—O1	97.45 (10)	F2—V1—N1	166.19 (11)
O1—V1—O2	112.52 (13)	F1—V1—N1	79.59 (10)
O1—V1—F2	100.53 (11)	V1—O1—Ag1	107.06 (11)
O2—V1—F2	100.10 (12)		

Symmetry code: (i) 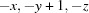.

**Table 2:** Hydrogen-bond geometry (Å, °)

*D*—H⋯*A*	*D*—H	H⋯*A*	*D*⋯*A*	*D*—H⋯*A*
O1*W*—H1*W*⋯F2^ii^	0.85	1.92	2.763 (4)	169
O1*W*—H2*W*⋯O2^iii^	0.85	2.08	2.910 (4)	166
O2*W*—H3*W*⋯O1*W*	0.85	2.08	2.753 (4)	136
O2*W*—H4*W*⋯O1	0.85	2.00	2.813 (4)	161
O3—H1*O*⋯O2*W* ^iv^	0.82	1.83	2.650 (4)	178
O5—H2*O*⋯F1^v^	0.82	1.77	2.589 (3)	178
C15—H15⋯F1^i^	0.93	2.19	2.966 (4)	141
C14—H14⋯F2^ii^	0.93	2.44	3.303 (4)	154

Symmetry codes: (i) 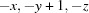; (ii) 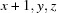; (iii) 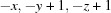; (iv) 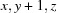; (v) 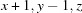.

**Table 3:** Experimental details

Crystal data	
Chemical formula	[Ag_2_V_2_F_4_O_4_(C_13_H_17_N_3_O_2_)_4_]·4H_2_O
*M*_r_	1518.86
Crystal system, space group	Triclinic, *P* 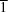
Temperature (K)	296
*a*, *b*, *c* (Å)	8.2673 (5), 12.6026 (6), 14.6757 (7)
α, β, γ (°)	77.985 (3), 86.535 (2), 83.945 (3)
*V* (Å^3^)	1486.05 (14)
*Z*	1
Radiation type	Mo *K*α
μ (mm^−1^)	1.05
Crystal size (mm)	0.26 × 0.12 × 0.10

Data collection	
Diffractometer	Bruker APEXII area-detector
Absorption correction	Numerical [face indexed (*SADABS*; Bruker, 2008 ▸)]
*T*_min_, *T*_max_	0.682, 0.890
No. of measured, independent and observed [*I* > 2σ(*I*)] reflections	25045, 7659, 4839
*R*_int_	0.053
(sin θ/λ)_max_ (Å^−1^)	0.676

Refinement	
*R*[*F* ^2^ > 2σ(*F* ^2^)], *wR*(*F* ^2^), *S*	0.045, 0.112, 1.06
No. of reflections	7659
No. of parameters	397
No. of restraints	1
H-atom treatment	H-atom parameters constrained
Δρ_max_, Δρ_min_ (e Å^−3^)	0.58, −0.70

Computer programs: *APEX2* and *SAINT* (Bruker, 2008 ▸), *SHELXS97* (Sheldrick, 2008 ▸), *SHELXL2014* (Sheldrick, 2015 ▸), *DIAMOND* (Brandenburg, 1999 ▸) and *WinGX* (Farrugia, 2012 ▸).

## supplementary crystallographic information

### Crystal data

**Table:**

[Ag_2_V_2_F_4_O_4_(C_13_H_17_N_3_O_2_)_4_]·4H_2_O	*Z* = 1
*M**_r_* = 1518.86	*F*(000) = 776
Triclinic, *P*1	*D*_x_ = 1.697 Mg m^−^^3^
*a* = 8.2673 (5) Å	Mo *K*α radiation, λ = 0.71073 Å
*b* = 12.6026 (6) Å	Cell parameters from 25045 reflections
*c* = 14.6757 (7) Å	θ = 1.7–28.7°
α = 77.985 (3)°	µ = 1.05 mm^−^^1^
β = 86.535 (2)°	*T* = 296 K
γ = 83.945 (3)°	Prism, light-yellow
*V* = 1486.05 (14) Å^3^	0.26 × 0.12 × 0.10 mm

### Data collection

**Table:**

Bruker APEXII area-detector diffractometer	4839 reflections with *I* > 2σ(*I*)
Radiation source: fine-focus sealed tube	*R*_int_ = 0.053
ω scans	θ_max_ = 28.7°, θ_min_ = 1.7°
Absorption correction: numerical [face indexed (SADABS; Bruker, 2008)]	*h* = −11→11
*T*_min_ = 0.682, *T*_max_ = 0.890	*k* = −16→17
25045 measured reflections	*l* = −19→19
7659 independent reflections	

### Refinement

**Table:**

Refinement on *F*^2^	Primary atom site location: structure-invariant direct methods
Least-squares matrix: full	Secondary atom site location: difference Fourier map
*R*[*F*^2^ > 2σ(*F*^2^)] = 0.045	Hydrogen site location: mixed
*wR*(*F*^2^) = 0.112	H-atom parameters constrained
*S* = 1.06	*w* = 1/[σ^2^(*F*_o_^2^) + (0.0428*P*)^2^ + 0.1791*P*] where *P* = (*F*_o_^2^ + 2*F*_c_^2^)/3
7659 reflections	(Δ/σ)_max_ = 0.001
397 parameters	Δρ_max_ = 0.58 e Å^−^^3^
1 restraint	Δρ_min_ = −0.70 e Å^−^^3^

### Special details

**Table:**

Geometry. All esds (except the esd in the dihedral angle between two l.s. planes) are estimated using the full covariance matrix. The cell esds are taken into account individually in the estimation of esds in distances, angles and torsion angles; correlations between esds in cell parameters are only used when they are defined by crystal symmetry. An approximate (isotropic) treatment of cell esds is used for estimating esds involving l.s. planes.

### Fractional atomic coordinates and isotropic or equivalent isotropic displacement parameters (Å^2^)

**Table:**

	*x*	*y*	*z*	*U*_iso_*/*U*_eq_	
Ag1	−0.00385 (4)	0.54697 (2)	0.11326 (2)	0.04628 (11)	
V1	−0.30899 (7)	0.54308 (4)	0.29331 (4)	0.03150 (14)	
F1	−0.3693 (3)	0.67346 (15)	0.21335 (14)	0.0467 (5)	
F2	−0.5052 (3)	0.49964 (18)	0.26987 (17)	0.0604 (6)	
O1	−0.1839 (3)	0.44852 (18)	0.26017 (19)	0.0480 (7)	
O2	−0.3264 (3)	0.5279 (2)	0.40630 (16)	0.0465 (6)	
O3	0.1863 (4)	1.1178 (2)	0.3251 (2)	0.0763 (10)	
H1O	0.1652	1.1836	0.3210	0.114*	
O4	0.2087 (4)	1.1221 (2)	0.4731 (2)	0.0697 (9)	
O5	0.7345 (4)	−0.1354 (2)	0.1879 (2)	0.0763 (10)	
H2O	0.7008	−0.1957	0.1971	0.114*	
O6	0.4929 (4)	−0.0858 (2)	0.1313 (2)	0.0754 (10)	
N1	−0.0998 (3)	0.6295 (2)	0.29829 (18)	0.0340 (6)	
N2	0.0147 (4)	0.6413 (2)	0.2265 (2)	0.0422 (7)	
N3	0.0900 (3)	0.7190 (2)	0.33460 (18)	0.0324 (6)	
N4	0.2080 (4)	0.4410 (2)	0.0703 (2)	0.0412 (7)	
N5	0.2061 (4)	0.4050 (2)	−0.0112 (2)	0.0444 (8)	
N6	0.4209 (4)	0.3254 (2)	0.06259 (18)	0.0356 (7)	
C1	−0.0521 (4)	0.6773 (3)	0.3618 (2)	0.0374 (8)	
H1	−0.1086	0.6817	0.4178	0.045*	
C2	0.1274 (5)	0.6938 (3)	0.2511 (2)	0.0425 (9)	
H2	0.2212	0.7114	0.2153	0.051*	
C3	0.1904 (4)	0.7734 (2)	0.3894 (2)	0.0290 (7)	
C4	0.1587 (5)	0.7283 (3)	0.4921 (2)	0.0385 (8)	
H4A	0.1819	0.6497	0.5054	0.046*	
H4B	0.0455	0.7457	0.5095	0.046*	
C5	0.2693 (5)	0.7795 (3)	0.5474 (3)	0.0496 (10)	
H5	0.2521	0.7500	0.6141	0.059*	
C6	0.4463 (5)	0.7555 (3)	0.5197 (3)	0.0600 (12)	
H6A	0.5146	0.7876	0.5560	0.072*	
H6B	0.4752	0.6773	0.5322	0.072*	
C7	0.4743 (4)	0.8014 (3)	0.4181 (3)	0.0475 (10)	
H7	0.5890	0.7852	0.4003	0.057*	
C8	0.3671 (4)	0.7488 (3)	0.3614 (3)	0.0472 (10)	
H8A	0.3852	0.7775	0.2954	0.057*	
H8B	0.3953	0.6706	0.3731	0.057*	
C9	0.1443 (4)	0.8964 (2)	0.3674 (2)	0.0352 (8)	
H9A	0.0301	0.9124	0.3837	0.042*	
H9B	0.1622	0.9251	0.3013	0.042*	
C10	0.2513 (4)	0.9494 (3)	0.4246 (3)	0.0380 (8)	
C11	0.2249 (5)	0.9011 (3)	0.5283 (2)	0.0461 (9)	
H11A	0.2915	0.9345	0.5646	0.055*	
H11B	0.1118	0.9165	0.5471	0.055*	
C12	0.4305 (4)	0.9237 (3)	0.3956 (3)	0.0415 (9)	
H12A	0.4475	0.9512	0.3292	0.050*	
H12B	0.4999	0.9590	0.4287	0.050*	
C13	0.2125 (5)	1.0721 (3)	0.4106 (3)	0.0514 (10)	
C14	0.3380 (5)	0.3922 (3)	0.1122 (2)	0.0423 (9)	
H14	0.3691	0.4026	0.1693	0.051*	
C15	0.3337 (5)	0.3364 (3)	−0.0129 (3)	0.0519 (10)	
H15	0.3613	0.2993	−0.0610	0.062*	
C16	0.5697 (4)	0.2489 (2)	0.0852 (2)	0.0328 (7)	
C17	0.6483 (5)	0.2732 (3)	0.1687 (3)	0.0447 (9)	
H17A	0.5725	0.2647	0.2223	0.054*	
H17B	0.6772	0.3476	0.1550	0.054*	
C18	0.8018 (5)	0.1935 (3)	0.1904 (3)	0.0467 (9)	
H18	0.8533	0.2085	0.2442	0.056*	
C19	0.7547 (5)	0.0767 (3)	0.2133 (2)	0.0421 (9)	
H19A	0.6806	0.0671	0.2675	0.050*	
H19B	0.8511	0.0263	0.2275	0.050*	
C20	0.6725 (4)	0.0529 (3)	0.1294 (2)	0.0376 (8)	
C21	0.5206 (4)	0.1337 (2)	0.1075 (2)	0.0337 (7)	
H21A	0.4677	0.1192	0.0547	0.040*	
H21B	0.4442	0.1251	0.1607	0.040*	
C22	0.6886 (5)	0.2630 (3)	0.0006 (3)	0.0471 (9)	
H22A	0.7184	0.3373	−0.0144	0.057*	
H22B	0.6376	0.2488	−0.0528	0.057*	
C23	0.8418 (5)	0.1830 (3)	0.0231 (3)	0.0516 (10)	
H23	0.9186	0.1920	−0.0308	0.062*	
C24	0.9208 (5)	0.2080 (3)	0.1068 (3)	0.0599 (12)	
H24A	1.0189	0.1592	0.1209	0.072*	
H24B	0.9507	0.2822	0.0923	0.072*	
C25	0.7922 (5)	0.0670 (3)	0.0448 (3)	0.0464 (9)	
H25A	0.8878	0.0156	0.0578	0.056*	
H25B	0.7414	0.0525	−0.0087	0.056*	
C26	0.6226 (5)	−0.0622 (3)	0.1493 (3)	0.0476 (9)	
O1W	0.2476 (3)	0.4641 (2)	0.4048 (2)	0.0595 (7)	
H1W	0.3296	0.4786	0.3682	0.089*	
H2W	0.2773	0.4552	0.4606	0.089*	
O2W	0.1186 (3)	0.33128 (19)	0.3067 (2)	0.0564 (7)	
H3W	0.1261	0.3502	0.3585	0.085*	
H4W	0.0243	0.3540	0.2866	0.085*	

### Atomic displacement parameters (Å^2^)

**Table:**

	*U*^11^	*U*^22^	*U*^33^	*U*^12^	*U*^13^	*U*^23^
Ag1	0.0503 (2)	0.05347 (19)	0.03972 (17)	0.00170 (14)	−0.00405 (13)	−0.02286 (13)
V1	0.0352 (3)	0.0265 (3)	0.0343 (3)	−0.0065 (2)	−0.0035 (2)	−0.0074 (2)
F1	0.0587 (14)	0.0416 (11)	0.0383 (12)	−0.0013 (10)	−0.0061 (10)	−0.0051 (9)
F2	0.0451 (14)	0.0697 (15)	0.0759 (17)	−0.0204 (11)	−0.0034 (12)	−0.0285 (13)
O1	0.0462 (16)	0.0342 (13)	0.0697 (18)	−0.0062 (11)	−0.0050 (13)	−0.0227 (12)
O2	0.0495 (17)	0.0532 (15)	0.0348 (14)	−0.0085 (12)	−0.0027 (12)	−0.0022 (12)
O3	0.118 (3)	0.0406 (16)	0.068 (2)	−0.0068 (17)	−0.011 (2)	−0.0057 (15)
O4	0.073 (2)	0.0575 (18)	0.091 (2)	−0.0061 (15)	−0.0108 (18)	−0.0400 (17)
O5	0.071 (2)	0.0330 (15)	0.122 (3)	−0.0048 (14)	−0.033 (2)	−0.0003 (16)
O6	0.087 (3)	0.0415 (16)	0.098 (3)	−0.0194 (16)	−0.042 (2)	0.0033 (16)
N1	0.0354 (16)	0.0372 (15)	0.0336 (15)	−0.0097 (12)	−0.0004 (13)	−0.0142 (12)
N2	0.0450 (19)	0.0518 (18)	0.0386 (17)	−0.0174 (14)	0.0052 (14)	−0.0247 (14)
N3	0.0341 (16)	0.0340 (14)	0.0333 (15)	−0.0084 (12)	0.0039 (12)	−0.0151 (12)
N4	0.0451 (19)	0.0443 (17)	0.0383 (17)	0.0040 (14)	−0.0067 (14)	−0.0205 (14)
N5	0.051 (2)	0.0509 (18)	0.0333 (16)	0.0055 (15)	−0.0092 (14)	−0.0176 (14)
N6	0.0435 (18)	0.0344 (15)	0.0306 (15)	0.0008 (13)	−0.0052 (13)	−0.0112 (12)
C1	0.033 (2)	0.048 (2)	0.0373 (19)	−0.0148 (16)	0.0065 (15)	−0.0199 (16)
C2	0.044 (2)	0.057 (2)	0.0333 (19)	−0.0212 (18)	0.0093 (16)	−0.0190 (17)
C3	0.0270 (17)	0.0314 (16)	0.0320 (17)	−0.0067 (13)	−0.0010 (13)	−0.0122 (13)
C4	0.045 (2)	0.0367 (18)	0.0353 (19)	−0.0130 (16)	−0.0025 (16)	−0.0052 (15)
C5	0.062 (3)	0.056 (2)	0.034 (2)	−0.022 (2)	−0.0089 (18)	−0.0061 (17)
C6	0.053 (3)	0.045 (2)	0.084 (3)	−0.0065 (19)	−0.035 (2)	−0.004 (2)
C7	0.026 (2)	0.047 (2)	0.074 (3)	−0.0029 (16)	−0.0016 (19)	−0.023 (2)
C8	0.034 (2)	0.045 (2)	0.071 (3)	−0.0073 (16)	0.0059 (19)	−0.0302 (19)
C9	0.036 (2)	0.0297 (17)	0.041 (2)	−0.0064 (14)	−0.0068 (15)	−0.0066 (14)
C10	0.0318 (19)	0.0296 (16)	0.055 (2)	−0.0063 (14)	−0.0107 (16)	−0.0106 (16)
C11	0.038 (2)	0.064 (2)	0.047 (2)	−0.0129 (18)	0.0019 (17)	−0.0321 (19)
C12	0.035 (2)	0.047 (2)	0.048 (2)	−0.0184 (16)	0.0010 (16)	−0.0160 (17)
C13	0.047 (2)	0.046 (2)	0.064 (3)	−0.0117 (18)	−0.009 (2)	−0.013 (2)
C14	0.049 (2)	0.045 (2)	0.036 (2)	0.0040 (17)	−0.0082 (17)	−0.0179 (16)
C15	0.059 (3)	0.062 (2)	0.038 (2)	0.014 (2)	−0.0126 (19)	−0.0252 (19)
C16	0.038 (2)	0.0290 (16)	0.0316 (18)	−0.0041 (14)	−0.0039 (15)	−0.0060 (13)
C17	0.048 (2)	0.040 (2)	0.051 (2)	−0.0030 (17)	−0.0113 (18)	−0.0186 (17)
C18	0.043 (2)	0.048 (2)	0.054 (2)	−0.0046 (17)	−0.0159 (19)	−0.0179 (18)
C19	0.047 (2)	0.043 (2)	0.0346 (19)	−0.0016 (17)	−0.0086 (17)	−0.0043 (16)
C20	0.048 (2)	0.0289 (17)	0.0357 (19)	−0.0026 (15)	−0.0043 (16)	−0.0056 (14)
C21	0.040 (2)	0.0335 (17)	0.0282 (17)	−0.0083 (14)	−0.0034 (15)	−0.0059 (14)
C22	0.051 (2)	0.040 (2)	0.045 (2)	−0.0083 (17)	0.0041 (19)	0.0047 (17)
C23	0.041 (2)	0.056 (2)	0.051 (2)	−0.0020 (19)	0.0122 (19)	−0.0001 (19)
C24	0.038 (2)	0.051 (2)	0.087 (3)	−0.0087 (19)	−0.007 (2)	−0.002 (2)
C25	0.050 (2)	0.043 (2)	0.044 (2)	0.0071 (17)	0.0032 (18)	−0.0120 (17)
C26	0.057 (3)	0.040 (2)	0.046 (2)	−0.0025 (19)	−0.0149 (19)	−0.0088 (17)
O1W	0.0571 (19)	0.0644 (18)	0.0593 (19)	−0.0086 (14)	−0.0060 (14)	−0.0151 (14)
O2W	0.0617 (19)	0.0372 (14)	0.0679 (19)	0.0009 (13)	−0.0066 (15)	−0.0072 (13)

### Geometric parameters (Å, º)

**Table:**

Ag1—N4	2.230 (3)	C8—H8A	0.9700
Ag1—N2	2.257 (3)	C8—H8B	0.9700
Ag1—N5^i^	2.262 (3)	C9—C10	1.543 (4)
Ag1—O1	2.700 (3)	C9—H9A	0.9700
V1—O1	1.627 (2)	C9—H9B	0.9700
V1—O2	1.628 (2)	C10—C13	1.519 (5)
V1—F2	1.839 (2)	C10—C11	1.529 (5)
V1—F1	1.850 (2)	C10—C12	1.536 (5)
V1—N1	2.152 (3)	C11—H11A	0.9700
O3—C13	1.288 (5)	C11—H11B	0.9700
O3—H1O	0.8200	C12—H12A	0.9700
O4—C13	1.214 (5)	C12—H12B	0.9700
O5—C26	1.301 (4)	C14—H14	0.9300
O5—H2O	0.8200	C15—H15	0.9300
O6—C26	1.201 (5)	C16—C21	1.513 (4)
N1—C1	1.309 (4)	C16—C17	1.521 (4)
N1—N2	1.368 (4)	C16—C22	1.528 (5)
N2—C2	1.306 (4)	C17—C18	1.539 (5)
N3—C2	1.339 (4)	C17—H17A	0.9700
N3—C1	1.342 (4)	C17—H17B	0.9700
N3—C3	1.496 (4)	C18—C24	1.519 (6)
N4—C14	1.301 (4)	C18—C19	1.526 (5)
N4—N5	1.367 (4)	C18—H18	0.9800
N5—C15	1.293 (4)	C19—C20	1.539 (5)
N5—Ag1^i^	2.262 (3)	C19—H19A	0.9700
N6—C15	1.334 (4)	C19—H19B	0.9700
N6—C14	1.335 (4)	C20—C26	1.514 (5)
N6—C16	1.489 (4)	C20—C25	1.533 (5)
C1—H1	0.9300	C20—C21	1.536 (5)
C2—H2	0.9300	C21—H21A	0.9700
C3—C8	1.512 (4)	C21—H21B	0.9700
C3—C4	1.513 (4)	C22—C23	1.540 (5)
C3—C9	1.529 (4)	C22—H22A	0.9700
C4—C5	1.531 (5)	C22—H22B	0.9700
C4—H4A	0.9700	C23—C25	1.525 (5)
C4—H4B	0.9700	C23—C24	1.528 (6)
C5—C11	1.509 (5)	C23—H23	0.9800
C5—C6	1.512 (6)	C24—H24A	0.9700
C5—H5	0.9800	C24—H24B	0.9700
C6—C7	1.496 (6)	C25—H25A	0.9700
C6—H6A	0.9700	C25—H25B	0.9700
C6—H6B	0.9700	O1W—H1W	0.8500
C7—C12	1.517 (5)	O1W—H2W	0.8500
C7—C8	1.535 (5)	O2W—H3W	0.8499
C7—H7	0.9800	O2W—H4W	0.8500
			
N4—Ag1—N2	121.35 (11)	C11—C10—C9	109.4 (3)
N4—Ag1—N5^i^	115.91 (10)	C12—C10—C9	108.4 (3)
N2—Ag1—N5^i^	120.21 (11)	C5—C11—C10	110.3 (3)
N4—Ag1—O1	114.33 (9)	C5—C11—H11A	109.6
N2—Ag1—O1	74.30 (9)	C10—C11—H11A	109.6
N5^i^—Ag1—O1	97.45 (10)	C5—C11—H11B	109.6
O1—V1—O2	112.52 (13)	C10—C11—H11B	109.6
O1—V1—F2	100.53 (11)	H11A—C11—H11B	108.1
O2—V1—F2	100.10 (12)	C7—C12—C10	109.5 (3)
O1—V1—F1	122.01 (12)	C7—C12—H12A	109.8
O2—V1—F1	122.74 (11)	C10—C12—H12A	109.8
F2—V1—F1	86.60 (10)	C7—C12—H12B	109.8
O1—V1—N1	87.04 (11)	C10—C12—H12B	109.8
O2—V1—N1	87.42 (12)	H12A—C12—H12B	108.2
F2—V1—N1	166.19 (11)	O4—C13—O3	123.5 (4)
F1—V1—N1	79.59 (10)	O4—C13—C10	123.6 (4)
V1—O1—Ag1	107.06 (11)	O3—C13—C10	112.9 (3)
C13—O3—H1O	109.5	N4—C14—N6	111.3 (3)
C26—O5—H2O	109.5	N4—C14—H14	124.3
C1—N1—N2	107.1 (3)	N6—C14—H14	124.3
C1—N1—V1	131.9 (2)	N5—C15—N6	111.9 (3)
N2—N1—V1	120.97 (19)	N5—C15—H15	124.0
C2—N2—N1	106.5 (3)	N6—C15—H15	124.0
C2—N2—Ag1	134.6 (2)	N6—C16—C21	108.4 (3)
N1—N2—Ag1	117.65 (19)	N6—C16—C17	109.7 (3)
C2—N3—C1	104.9 (3)	C21—C16—C17	110.0 (3)
C2—N3—C3	127.8 (3)	N6—C16—C22	108.9 (3)
C1—N3—C3	127.2 (3)	C21—C16—C22	109.9 (3)
C14—N4—N5	106.5 (3)	C17—C16—C22	110.0 (3)
C14—N4—Ag1	133.2 (2)	C16—C17—C18	108.7 (3)
N5—N4—Ag1	119.9 (2)	C16—C17—H17A	110.0
C15—N5—N4	106.3 (3)	C18—C17—H17A	110.0
C15—N5—Ag1^i^	129.4 (2)	C16—C17—H17B	110.0
N4—N5—Ag1^i^	124.1 (2)	C18—C17—H17B	110.0
C15—N6—C14	103.9 (3)	H17A—C17—H17B	108.3
C15—N6—C16	125.8 (3)	C24—C18—C19	109.8 (3)
C14—N6—C16	130.2 (3)	C24—C18—C17	109.7 (3)
N1—C1—N3	110.4 (3)	C19—C18—C17	109.5 (3)
N1—C1—H1	124.8	C24—C18—H18	109.3
N3—C1—H1	124.8	C19—C18—H18	109.3
N2—C2—N3	111.0 (3)	C17—C18—H18	109.3
N2—C2—H2	124.5	C18—C19—C20	109.5 (3)
N3—C2—H2	124.5	C18—C19—H19A	109.8
N3—C3—C8	107.8 (2)	C20—C19—H19A	109.8
N3—C3—C4	108.5 (2)	C18—C19—H19B	109.8
C8—C3—C4	110.8 (3)	C20—C19—H19B	109.8
N3—C3—C9	109.8 (3)	H19A—C19—H19B	108.2
C8—C3—C9	109.8 (3)	C26—C20—C25	109.0 (3)
C4—C3—C9	110.1 (3)	C26—C20—C21	109.1 (3)
C3—C4—C5	108.1 (3)	C25—C20—C21	109.3 (3)
C3—C4—H4A	110.1	C26—C20—C19	111.2 (3)
C5—C4—H4A	110.1	C25—C20—C19	109.0 (3)
C3—C4—H4B	110.1	C21—C20—C19	109.1 (3)
C5—C4—H4B	110.1	C16—C21—C20	109.5 (3)
H4A—C4—H4B	108.4	C16—C21—H21A	109.8
C11—C5—C6	109.7 (3)	C20—C21—H21A	109.8
C11—C5—C4	108.6 (3)	C16—C21—H21B	109.8
C6—C5—C4	110.9 (3)	C20—C21—H21B	109.8
C11—C5—H5	109.2	H21A—C21—H21B	108.2
C6—C5—H5	109.2	C16—C22—C23	109.2 (3)
C4—C5—H5	109.2	C16—C22—H22A	109.8
C7—C6—C5	109.6 (3)	C23—C22—H22A	109.8
C7—C6—H6A	109.7	C16—C22—H22B	109.8
C5—C6—H6A	109.7	C23—C22—H22B	109.8
C7—C6—H6B	109.7	H22A—C22—H22B	108.3
C5—C6—H6B	109.7	C25—C23—C24	110.7 (3)
H6A—C6—H6B	108.2	C25—C23—C22	108.8 (3)
C6—C7—C12	111.3 (3)	C24—C23—C22	108.8 (3)
C6—C7—C8	109.2 (3)	C25—C23—H23	109.5
C12—C7—C8	108.2 (3)	C24—C23—H23	109.5
C6—C7—H7	109.3	C22—C23—H23	109.5
C12—C7—H7	109.3	C18—C24—C23	109.4 (3)
C8—C7—H7	109.3	C18—C24—H24A	109.8
C3—C8—C7	109.2 (3)	C23—C24—H24A	109.8
C3—C8—H8A	109.8	C18—C24—H24B	109.8
C7—C8—H8A	109.8	C23—C24—H24B	109.8
C3—C8—H8B	109.8	H24A—C24—H24B	108.2
C7—C8—H8B	109.8	C23—C25—C20	109.5 (3)
H8A—C8—H8B	108.3	C23—C25—H25A	109.8
C3—C9—C10	108.1 (3)	C20—C25—H25A	109.8
C3—C9—H9A	110.1	C23—C25—H25B	109.8
C10—C9—H9A	110.1	C20—C25—H25B	109.8
C3—C9—H9B	110.1	H25A—C25—H25B	108.2
C10—C9—H9B	110.1	O6—C26—O5	122.0 (4)
H9A—C9—H9B	108.4	O6—C26—C20	124.2 (4)
C13—C10—C11	107.8 (3)	O5—C26—C20	113.8 (3)
C13—C10—C12	109.3 (3)	H1W—O1W—H2W	108.4
C11—C10—C12	109.1 (3)	H3W—O2W—H4W	108.4
C13—C10—C9	112.9 (3)		
			
O2—V1—O1—Ag1	−138.31 (12)	C11—C10—C13—O4	19.2 (5)
F2—V1—O1—Ag1	116.01 (11)	C12—C10—C13—O4	−99.3 (5)
F1—V1—O1—Ag1	23.43 (16)	C9—C10—C13—O4	140.1 (4)
N1—V1—O1—Ag1	−52.34 (11)	C11—C10—C13—O3	−161.8 (3)
C1—N1—N2—C2	1.2 (4)	C12—C10—C13—O3	79.8 (4)
V1—N1—N2—C2	−178.1 (2)	C9—C10—C13—O3	−40.9 (5)
C1—N1—N2—Ag1	170.6 (2)	N5—N4—C14—N6	−0.5 (4)
V1—N1—N2—Ag1	−8.7 (3)	Ag1—N4—C14—N6	171.8 (2)
C14—N4—N5—C15	0.5 (4)	C15—N6—C14—N4	0.2 (4)
Ag1—N4—N5—C15	−173.0 (3)	C16—N6—C14—N4	−175.9 (3)
C14—N4—N5—Ag1^i^	176.9 (2)	N4—N5—C15—N6	−0.4 (5)
Ag1—N4—N5—Ag1^i^	3.4 (4)	Ag1^i^—N5—C15—N6	−176.5 (2)
N2—N1—C1—N3	−0.5 (4)	C14—N6—C15—N5	0.1 (5)
V1—N1—C1—N3	178.6 (2)	C16—N6—C15—N5	176.5 (3)
C2—N3—C1—N1	−0.3 (4)	C15—N6—C16—C21	−68.7 (4)
C3—N3—C1—N1	−175.8 (3)	C14—N6—C16—C21	106.7 (4)
N1—N2—C2—N3	−1.4 (4)	C15—N6—C16—C17	171.2 (4)
Ag1—N2—C2—N3	−168.2 (2)	C14—N6—C16—C17	−13.4 (5)
C1—N3—C2—N2	1.1 (4)	C15—N6—C16—C22	50.7 (5)
C3—N3—C2—N2	176.5 (3)	C14—N6—C16—C22	−133.8 (4)
C2—N3—C3—C8	−26.6 (4)	N6—C16—C17—C18	−179.8 (3)
C1—N3—C3—C8	147.8 (3)	C21—C16—C17—C18	61.1 (4)
C2—N3—C3—C4	−146.7 (3)	C22—C16—C17—C18	−60.0 (4)
C1—N3—C3—C4	27.8 (4)	C16—C17—C18—C24	60.2 (4)
C2—N3—C3—C9	92.9 (4)	C16—C17—C18—C19	−60.4 (4)
C1—N3—C3—C9	−92.6 (4)	C24—C18—C19—C20	−60.6 (4)
N3—C3—C4—C5	176.8 (3)	C17—C18—C19—C20	59.9 (4)
C8—C3—C4—C5	58.6 (4)	C18—C19—C20—C26	−179.6 (3)
C9—C3—C4—C5	−63.0 (4)	C18—C19—C20—C25	60.2 (4)
C3—C4—C5—C11	62.2 (4)	C18—C19—C20—C21	−59.2 (4)
C3—C4—C5—C6	−58.4 (4)	N6—C16—C21—C20	179.0 (2)
C11—C5—C6—C7	−59.7 (4)	C17—C16—C21—C20	−61.1 (4)
C4—C5—C6—C7	60.2 (4)	C22—C16—C21—C20	60.1 (4)
C5—C6—C7—C12	59.4 (4)	C26—C20—C21—C16	−178.7 (3)
C5—C6—C7—C8	−60.1 (4)	C25—C20—C21—C16	−59.6 (3)
N3—C3—C8—C7	−178.7 (3)	C19—C20—C21—C16	59.6 (4)
C4—C3—C8—C7	−60.1 (4)	N6—C16—C22—C23	−179.2 (3)
C9—C3—C8—C7	61.7 (4)	C21—C16—C22—C23	−60.6 (4)
C6—C7—C8—C3	60.1 (4)	C17—C16—C22—C23	60.5 (4)
C12—C7—C8—C3	−61.3 (4)	C16—C22—C23—C25	60.5 (4)
N3—C3—C9—C10	−179.6 (3)	C16—C22—C23—C24	−60.2 (4)
C8—C3—C9—C10	−61.2 (4)	C19—C18—C24—C23	59.4 (4)
C4—C3—C9—C10	61.0 (3)	C17—C18—C24—C23	−61.0 (4)
C3—C9—C10—C13	−178.2 (3)	C25—C23—C24—C18	−58.8 (4)
C3—C9—C10—C11	−58.2 (3)	C22—C23—C24—C18	60.7 (4)
C3—C9—C10—C12	60.6 (3)	C24—C23—C25—C20	59.0 (4)
C6—C5—C11—C10	60.2 (4)	C22—C23—C25—C20	−60.6 (4)
C4—C5—C11—C10	−61.2 (4)	C26—C20—C25—C23	179.3 (3)
C13—C10—C11—C5	−177.3 (3)	C21—C20—C25—C23	60.1 (4)
C12—C10—C11—C5	−58.8 (4)	C19—C20—C25—C23	−59.1 (4)
C9—C10—C11—C5	59.6 (4)	C25—C20—C26—O6	−104.9 (5)
C6—C7—C12—C10	−58.3 (4)	C21—C20—C26—O6	14.5 (5)
C8—C7—C12—C10	61.7 (4)	C19—C20—C26—O6	134.9 (4)
C13—C10—C12—C7	174.6 (3)	C25—C20—C26—O5	74.7 (4)
C11—C10—C12—C7	57.0 (4)	C21—C20—C26—O5	−166.0 (3)
C9—C10—C12—C7	−62.0 (4)	C19—C20—C26—O5	−45.5 (5)

Symmetry code: (i) −*x*, −*y*+1, −*z*.

### Hydrogen-bond geometry (Å, º)

**Table:**

*D*—H···*A*	*D*—H	H···*A*	*D*···*A*	*D*—H···*A*
O1*W*—H1*W*···F2^ii^	0.85	1.92	2.763 (4)	169
O1*W*—H2*W*···O2^iii^	0.85	2.08	2.910 (4)	166
O2*W*—H3*W*···O1*W*	0.85	2.08	2.753 (4)	136
O2*W*—H4*W*···O1	0.85	2.00	2.813 (4)	161
O3—H1*O*···O2*W*^iv^	0.82	1.83	2.650 (4)	178
O5—H2*O*···F1^v^	0.82	1.77	2.589 (3)	178
C15—H15···F1^i^	0.93	2.19	2.966 (4)	141
C14—H14···F2^ii^	0.93	2.44	3.303 (4)	154

Symmetry codes: (i) −*x*, −*y*+1, −*z*; (ii) *x*+1, *y*, *z*; (iii) −*x*, −*y*+1, −*z*+1; (iv) *x*, *y*+1, *z*; (v) *x*+1, *y*−1, *z*.
